# Supplementary figures and images for: Effects of Vocational Rehabilitation on the Use of Health Care Services in Finland: A Propensity Score Analysis
Source: Int J Environ Res Public Health. 2022 Nov 28;19(23):15809. doi: 10.3390/ijerph192315809 (PMC9735791; doi:10.3390/ijerph192315809)

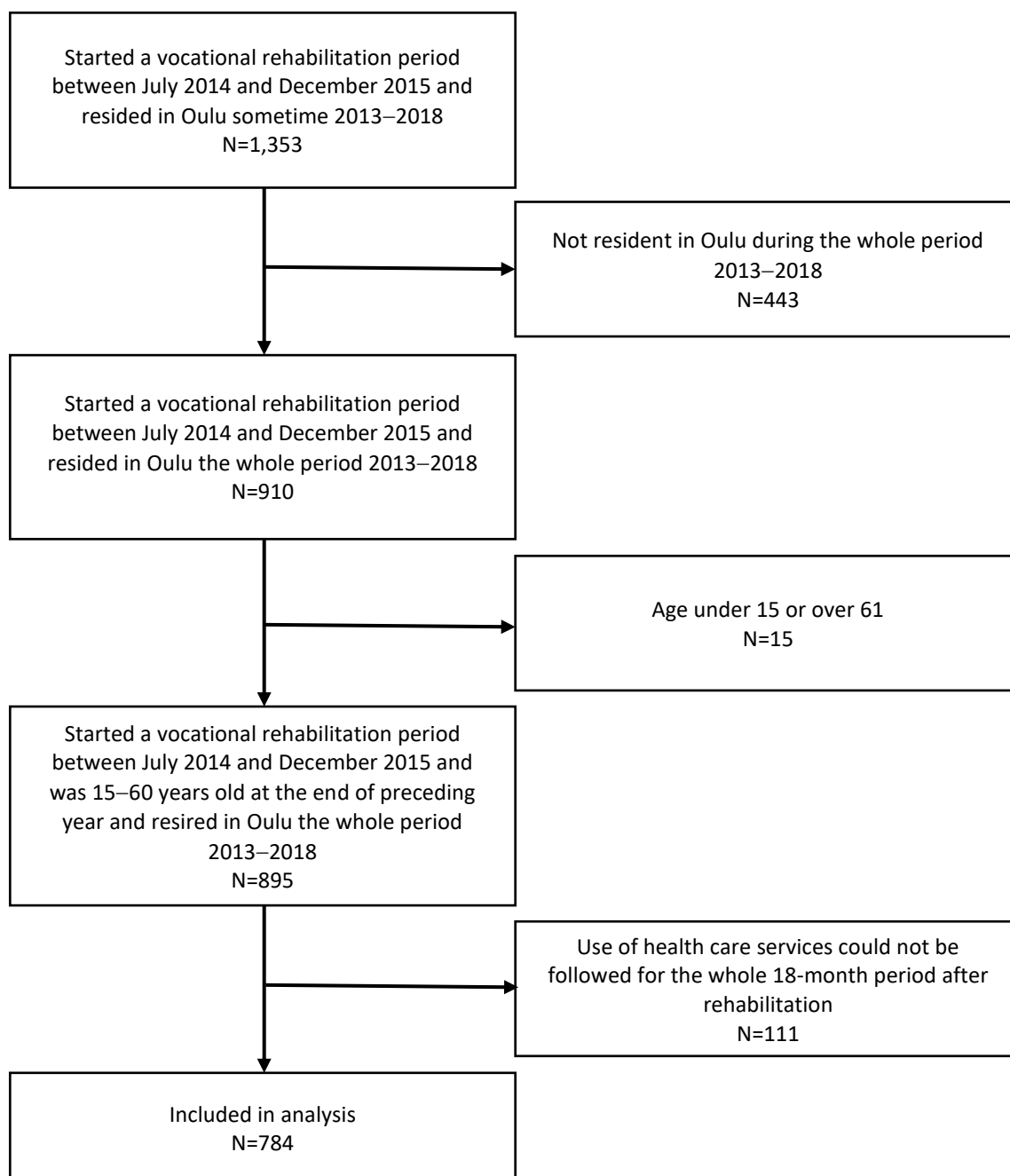

**Supplementary Figure S1. Flowchart of subjects who met inclusion criteria.**

Supplement: Supplementary file 1 [file ijerph-19-15809-s001.zip › ijerph-2014380-supplementary.pdf]
